# Personalised nutrition advice reduces intake of discretionary foods and beverages: findings from the Food4Me randomised controlled trial

**DOI:** 10.1186/s12966-021-01136-5

**Published:** 2021-06-07

**Authors:** Katherine M. Livingstone, Carlos Celis-Morales, Santiago Navas-Carretero, Rodrigo San-Cristobal, Hannah Forster, Clara Woolhead, Clare B. O’Donovan, George Moschonis, Yannis Manios, Iwona Traczyk, Thomas E. Gundersen, Christian A. Drevon, Cyril F. M. Marsaux, Rosalind Fallaize, Anna L. Macready, Hannelore Daniel, Wim H. M. Saris, Julie A. Lovegrove, Mike Gibney, Eileen R. Gibney, Marianne Walsh, Lorraine Brennan, J. Alfredo Martinez, John C. Mathers, on behalf of the Food4Me Study

**Affiliations:** 1 Human Nutrition Research Centre, Population Health Sciences Institute, Newcastle University, William Leech Building, Newcastle upon Tyne, NE2 4HH UK; 2 Institute for Physical Activity and Nutrition, School of Exercise and Nutrition Sciences, Deakin University, Geelong, 3220 VIC Australia; 3 BHF Glasgow Cardiovascular Research Centre, Institute of Cardiovascular and Medical Sciences, University of Glasgow, Glasgow, UK; 4 Research Unit on Education, Physical Activity and Health (GEEAFyS), Universidad Católica del Maule, Talca, Chile; 5 Centre of Research in Exercise Physiology (CIFE), Universidad Mayor, Santiago, Chile; 6 Department of Nutrition, Food Science and Physiology, University of Navarra, Pamplona, Spain; 7 CIBERobn, Fisiopatología de la Obesidad y Nutrición, Instituto de Salud Carlos III, Madrid, Spain; 8 Precision Nutrition and Cardiometabolic Health, IMDEA-Food Institute (Madrid Institute for Advanced Studies), CEI UAM + CSIC, Madrid, Spain; 9 UCD Institute of Food and Health, University College Dublin, Belfield, Dublin 4 Republic of Ireland; 10 Department of Nutrition and Dietetics, Harokopio University, Athens, Greece; 11 Department of Dietetics, Nutrition and Sport, School of Allied Health, Human Services and Sport, La Trobe University, Bundoora, 3086 VIC Australia; 12 Department of Human Nutrition, Faculty of Health Sciences, Medical University of Warsaw, Warsaw, Poland; 13 Vitas AS, Gaustadalléen 21, 0349 Oslo, Norway; 14 Department of Nutrition, Faculty of Medicine, Institute of Basic Medical Sciences, University of Oslo, Oslo, Norway; 15 Department of Human Biology, NUTRIM, School for Nutrition and Translational Research in Metabolism, Maastricht University Medical Centre, Maastricht, The Netherlands; 16 Department of Food and Nutritional Sciences, Hugh Sinclair Unit of Human Nutrition and Institute for Cardiovascular and Metabolic Research, University of Reading, Reading, UK; 17 School of Life and Medical Sciences, University of Hertfordshire, Hatfield, UK; 18 Molecular Nutrition Unit, Department Food and Nutrition, Technische Universität München, München, Germany

**Keywords:** Discretionary, Discretionary foods and beverages, Personalised nutrition, Internet-based, Intervention, European, Adults, Food4Me

## Abstract

**Background:**

The effect of personalised nutrition advice on discretionary foods intake is unknown. To date, two national classifications for discretionary foods have been derived. This study examined changes in intake of discretionary foods and beverages following a personalised nutrition intervention using these two classifications.

**Methods:**

Participants were recruited into a 6-month RCT across seven European countries (Food4Me) and were randomised to receive generalised dietary advice (control) or one of three levels of personalised nutrition advice (based on diet [L1], phenotype [L2] and genotype [L3]). Dietary intake was derived from an FFQ. An analysis of covariance was used to determine intervention effects at month 6 between personalised nutrition (overall and by levels) and control on i) percentage energy from discretionary items and ii) percentage contribution of total fat, SFA, total sugars and salt to discretionary intake, defined by Food Standards Scotland (FSS) and Australian Dietary Guidelines (ADG) classifications.

**Results:**

Of the 1607 adults at baseline, *n* = 1270 (57% female) completed the intervention. Percentage sugars from FSS discretionary items was lower in personalised nutrition vs control (19.0 ± 0.37 vs 21.1 ± 0.65; *P* = 0.005). Percentage energy (31.2 ± 0.59 vs 32.7 ± 0.59; *P* = 0.031), percentage total fat (31.5 ± 0.37 vs 33.3 ± 0.65; *P* = 0.021), SFA (36.0 ± 0.43 vs 37.8 ± 0.75; *P* = 0.034) and sugars (31.7 ± 0.44 vs 34.7 ± 0.78; *P* < 0.001) from ADG discretionary items were lower in personalised nutrition vs control. There were greater reductions in ADG percentage energy and percentage total fat, SFA and salt for those randomised to L3 vs L2.

**Conclusions:**

Compared with generalised dietary advice, personalised nutrition advice achieved greater reductions in discretionary foods intake when the classification included all foods high in fat, added sugars and salt. Future personalised nutrition approaches may be used to target intake of discretionary foods.

**Trial registration:**

Clinicaltrials.gov NCT01530139. Registered 9 February 2012.

**Supplementary Information:**

The online version contains supplementary material available at 10.1186/s12966-021-01136-5.

## Background

The consumption of discretionary foods and beverages is discouraged in most dietary guidelines internationally [1–3]. While discretionary foods and beverages are broadly defined as being high in fat, added sugars and salt [3], there are inconsistencies between nutrient- and food-based approaches in dietary guidelines. Moreover, few European countries have provided a clear definition of which foods and beverages constitute discretionary foods. In the UK, Food Standards Scotland (FSS) has classified discretionary foods as confectionery, sweet biscuits, crisps, savoury snacks, cakes, sweet pastries, puddings and sugar containing soft drinks. Other energy-dense foods, such as processed meats and burgers, are not classified as discretionary by FSS as they provide nutrients and are typically consumed as a part of a meal [4]. In contrast, internationally, the classification of discretionary foods in the 2013 Australian Dietary Guidelines (ADG) does not make this distinction and includes all foods and beverages high in fat, added sugars and/or salt, including processed meats and alcoholic beverages, which are disregarded in the FSS classification [3, 5]. Similarly, the Dietary Guidelines for Americans 2015–2020 discourage calories from all foods and beverages high in added sugars, saturated fat, sodium, as well as alcoholic beverages [6]. These two approaches to classifying discretionary foods, based solely on energy density and based solely on snack foods, create challenges for comparing and reducing population intake of these foods. The increasing globalisation and politicisation of the food system, including the degree of food processing [7], suggest that there is a need to understand the utility of the classification “discretionary foods” when designing dietary interventions that aim to reduce intake of foods associated with poorer health.

Although, there is limited data on the percentage contribution of discretionary foods in Europe, recent national surveys suggest that discretionary foods and beverages in adults contribute up to 19% of daily energy intake in Scotland [4], and globally, up to 33, 28 and 26% in Australia [8], US [9], and Mexico [10], respectively. Given the poor nutritional profile of many discretionary foods and beverages, and that consumption of these foods is associated with greater risk of cardiovascular disease, obesity and all-cause mortality [11–15], there is a need to reduce intake of discretionary foods and beverages. Efforts to reduce discretionary food and beverage intake have typically focused on one-size-fits-all dietary recommendations [16, 17]. Increasingly, interventions are being designed and implemented using behavioural advice tailored to characteristics of the target population [18]. Personalised nutrition interventions leverage human individuality to design nutrition strategies to optimize health [19]. Emerging evidence suggests that personalised nutrition approaches offer an alternative and potentially more effective strategy to improve dietary intake compared with generalised dietary advice [20–22]. This includes successfully reducing intake of some discretionary foods and associated nutrients, such as red meat and salt intake [21, 23]. However, the effectiveness of personalised nutrition interventions for reducing intake of all discretionary foods and beverages remains unclear.

Research on reducing intake of discretionary foods and beverages is limited by lack of consensus in how to define and to classify discretionary food items. Moreover, no studies have provided a pan-European perspective on the effectiveness of personalised nutrition intervention to reduce intake of these foods. The Food4Me Study was a 6-month personalised nutrition intervention conducted across seven European countries that showed that personalised nutrition advice improved dietary intakes more than generalised dietary advice [18, 24–26]. The present study is a secondary analysis of the Food4Me study aiming to examine changes in intake of discretionary foods and beverages after 3 and 6 months of intervention using both the FSS and ADG classifications.

## Methods

### Study design

The Food4Me Study [27] was a 6-month, 4-arm, internet-based RCT conducted in seven European countries, designed to compare the effects of personalised dietary and physical activity advice with generalised advice in changing dietary and lifestyle behaviors [18, 23, 25, 28, 29]. Recruitment included newspapers, radio advertisements and flyers and potential participants could register their details via the Food4Me website [27]. Participants were asked via email to complete online questionnaires and to provide biological samples at baseline and after 3 and 6 months intervention. Participants could interact via email with the dietitians, nutritionists and researchers at each center during the 6-month intervention. Participants were randomised to one of four intervention arms and received either non-personalised, generalised dietary advice (control; Level 0), or one of three levels of personalised nutrition based on individual dietary, physical activity, phenotypic and genotypic data (see below). A total of 17 behaviour change techniques were embedded in the intervention design, including motivation behaviours and self-regulatory capacity or skill-related behaviours and promotion of supporting activities [18, 30]. Participants were asked to complete an online food frequency questionnaire (FFQ), the Baecke physical activity questionnaire [31], to wear accelerometers and to provide self-measured anthropometric information, buccal swabs and dry blood spot cards. Participants (*n* = 1607) were recruited between August 2012 and August 2013. Each university or research centre delivering the intervention obtained Research Ethics Committee approval for the study from their relevant local or national committee. The Food4Me trial was registered as an RCT (NCT01530139) at wwww.clinicaltrials.gov. Participants signed online consent forms [18]. The Consolidated Standards of Reporting Trials (CONSORT) 2010 checklist was used for reporting this study (see Additional File 1).

### Eligibility criteria

Participants aged ≥18 years were included. The following exclusion criteria were applied: (i) pregnant or lactating; (ii) no or limited access to the Internet; (iii) following a prescribed diet for any reason, including weight loss, in the last 3 months; (iv) diabetes, coeliac disease, Crohn’s disease, or any metabolic disease or condition altering nutritional requirements.

### Randomisation and masking

An urn randomisation scheme was used to allocate individuals to each treatment arm (see Additional File 2 for TIDieR checklist for intervention description). Participants randomised to Level 1 (L1) received personalised dietary advice based on current diet and physical activity alone, Level 2 (L2) received personalised dietary advice based on dietary, physical activity﻿ and phenotypic data and Level 3 (L3) received personalised dietary advice based on dietary, physical activity﻿, phenotypic and genotypic data. Personalised dietary feedback was based on how intake of specific nutrients compared with recommended intakes, which was then translated into advice on changing intake of food groups (fruits and vegetables, whole grain products, fish, dairy products and meat). Personalised phenotypic feedback was based on anthropometric measurements and nutrient- and metabolic-related biomarkers and specific variants in five nutrient-responsive genes were used to provide personalised genotypic feedback. Personalised advice on physical activity was based on accelerometer data and responses to the Baecke Questionnaire.

Participants randomised to the control group (L0) received dietary advice based on population-level healthy eating guidelines. This non-personalised dietary advice was derived from national dietary recommendations in each of the seven European countries and included generalised advice on the food groups listed above. In addition, these recommendations included a generic physical activity﻿ recommendation. Further details of the Food4Me study are provided elsewhere [18].

### Personalised feedback report

Participants randomised to L1, L2 and L3 received personalised feedback reports via email at baseline and at months 3 and 6 of the intervention. For those randomised to L1, L2 and L3, algorithms were used to provide participants with three specific top priority food-based dietary goals (e.g. designed to reduce intake of total fat, saturated fat, carbohydrate and salt) according to the individual’s intake of foods and nutrients [32]. Dietary advice was not given based on discretionary food intake per se, although messaging often referred to specific discretionary foods as targets to swap for healthier options. For example, if salt intake was identified as a top nutrient to change and meat-based dishes were the main contributing food sources, then a message may include “Reduce your intake of processed meats and pies; swap salami, ham and bacon for turkey or beef.” Added sugars were not a target nutrient, although advice on table sugar, honey, soft drinks and confectionary was given if intake of carbohydrates were identified as a top target nutrient. For participants randomised to L2 and L3, the dietary advice was also based on phenotypic data (L2) and phenotypic plus genotypic data (L3) [18]. Phenotypic data used to derive feedback included circulating concentrations of fasting blood cholesterol glucose, omega-3 and carotenoids, as well as BMI and waist circumference, while genotypic data included *FTO*, *FADS1*, *TCF7L2*, *APOE(e4)* and *MTHFR*. For example, a participant randomised to L3 with a high saturated fat intake from meat-based dishes, who also had high cholesterol and carried the *APOE* genetic risk variant, could receive the message “You have a genetic variation that can benefit by keeping a healthy intake of saturated fat and a normal level of blood cholesterol. Swap savoury pies and processed meats e.g., burgers, sausages and chicken goujons for lean meats or skinless chicken breast.”

### Dietary measures

Participants completed an online semi-quantitative FFQ to estimate dietary intake at baseline and at months 3 and 6 of the intervention. The FFQ was developed and validated for the Food4Me Study [33, 34] and included 157 food items consumed frequently in each of the seven recruitment countries. FFQs were available in the language of the country, with additional country-specific foods added to the FFQ for the Netherlands, Germany, Greece, Spain, Poland (e.g., ‘stroopwafels’ and ‘baklava’ were added to the Dutch and Greek FFQ, respectively). Nutritional composition and portion sizes were calculated from the 2008–2010 National Adult Nutrition Survey database [35], with relevant national food composition databases used for foods unique to specific countries. Further information on the development of the FFQ is detailed elsewhere [33, 36].

Two measures of discretionary foods and beverages intake were created by identifying relevant food and beverage items from the Food4Me FFQ, aligned to the FSS and ADG classification of discretionary foods. Using the FSS classification of discretionary foods and beverages [4], a total of 22 FFQ items were included: sweet biscuits (2 items), cakes, pastries and puddings (8 items), ice cream and desserts (2 items), confectionary (3 items), crisps and savoury snacks (4 items) and sugar containing drinks (3 items). The second measure, AGD discretionary, included a total of 59 FFQ items which, in addition to those food items within the FSS discretionary classification, also included savoury pastries and pies, processed meats, burgers and sausages (10 items), commercially fried foods (4 items), fatty and/or salty snack foods (2 items), cream, butter and spreads which are high in saturated fats or sugars (15 items) and alcoholic drinks or other beverages (6 items). As the ADG classification also includes criteria based on nutrient profiles at the food code level, decisions on the classification of FFQ food groups were made to align as best as possible, e.g. for burgers, discretionary foods are defined as those with > 5 g saturated fat per 100 g as per the ADG classification. With no differentiation between burgers available in the Food4Me FFQ, all burgers were included in the ADG discretionary classification. A full list of the foods and beverages included in each of the two measures is provided in Additional File 3.

The total percentage of energy (%E) from FSS and AGD discretionary foods and beverages and the percentage contribution of total fat, SFA and total sugars to FSS and AGD discretionary foods and beverages were estimated. Percentage energy from discretionary foods/beverages was calculated by first dividing the energy composition (kJ/ 100 g) of each discretionary FFQ item by 100, multiplying the grams consumed by the energy content per gram, summing for all discretionary food items, then dividing the discretionary energy intake by the total energy intake and multiplying by 100. For example, for a participant with a total energy intake of 9000 kJ/ day consuming two 20 g chocolate biscuits (providing 2053 kJ/ 100 g) per day and no other discretionary items, the contribution of discretionary foods (821 kJ) to their daily energy intake would be 9%. Similarly, to calculate the contribution to total intake of nutrients as a percentage, the total fat, SFA, sugars and salt composition (g/ 100 g) of each discretionary FFQ item was divided by 100, the number of grams consumed was multiplied by the nutrient content per gram, and summed for all discretionary foods. The discretionary nutrient intake was then divided by the total nutrient intake and multiplied by 100. One of the food groups included in personalised nutrition advice was sweets and snacks (21 FFQ food items – see Additional File 3). Given that the largest overlap between the FSS and ADG discretionary food classifications was in sweets and snacks, the contributions of total fat, SFA and total sugars made by sweets and snacks were also calculated. Energy misreporting was estimated as a binary variable (yes, no): under-reporting was operationalized as energy intake less than basal metabolic rate*1.1 [37], where basal metabolic rate was calculated according to the Oxford equation [38] and over-reporting as more than 4500 kcal/day [39].

Participants completed a dietary change questionnaire at months 3 and 6 of the intervention. This questionnaire asked whether participants agreed with the following statement: “Over the past month, since receiving my dietary feedback I have made changes to reduce the amount of fat I eat”, and equivalent questions were asked for sugar and salt. Responses included “strongly disagree”, “disagree”, “neither disagree nor agree”, “agree” and “strongly agree”. Three binary variables were created to reflect participants’ perceptions of reduction in intake of fat, sugar and salt; participants who selected “strongly agree” or “agree” at month 3 and/or month 6 were classified as perceiving a reduced consumption of that nutrient. A binary composite measure of perceived reduction in intake of any of these three nutrients (fat, sugar and/or salt) over the 6 month intervention was created by aggregating all participants who were classified as perceiving a reduced consumption of any of the three nutrient at month 3 and/or month 6.

### Study variables

Participants self-reported smoking habits and occupations. Country of residence was treated as a dummy variable. Physical activity level, moderate to vigorous physical activity (min/day), sedentary time (min/day) and the percentage of individuals meeting physical activity recommendations (> 150 min moderate physical activity or > 75 min vigorous physical activity or an equivalent combination of moderate and vigorous physical activity per week in bouts of at least 10 min [40]) were estimated from triaxial accelerometers (TracmorD, Philips Consumer Lifestyle.

Body weight (kg), height (m) and waist circumference (WC; cm) were self-measured and self-reported. Body mass index (BMI; kg/m^2^) was estimated from body weight and height. Participants were classified as overweight/obese, or not, using standard WHO classifications [41]. Self-reported anthropometric measurements were validated in a sub-sample of the participants (*n* = 140) and showed a high degree of correlation between self-reported and measures values [42].

### Statistical analysis

All statistical analyses were performed using Stata (version 16; StataCorp, College Station, TX, USA). Details on the sample size for the Food4Me Study are provided elsewhere [18, 23]. Complete case analysis was used to handle missing data. Given that adjustment for multiple comparisons may increase the risk of type 2 error [43], no adjustment for multiple comparisons was included. Mean and SD and percentages were used to present continuous and categorical variables, respectively. Percentage energy (%E) from discretionary items (using the FSS classification) were recoded into quartiles for descriptive purposes only. Multiple regression analyses were used to examine trends in participant characteristics (dependant variables) across quartiles of % E from discretionary items (independent variable). Linear and logistic regression analyses were used for continuous (age, body weight, BMI, WC, physical activity level, moderate/vigorous physical activity, sedentary time, total cholesterol concentrations) and categorical (occupation, weight status, meeting physical activity recommendation, smoking, medication use and energy misreporting) outcome variables, respectively. Trend analyses were adjusted for age, sex and country. Physical activity level, moderate to vigorous activity and sedentary time were additionally adjusted for time wearing the accelerometer and for season. Pearson’s correlation was used to examine the correlation between FSS and ADG measures for %E from discretionary items.

To examine change in intake of discretionary foods and beverages at month 3 and month 6 according to intervention arm, we used an analysis of covariance with baseline intake as a covariate. Three contrasts were used to compare change between intervention arms. Contrast 1 compared L0 (control) with the mean of L1-L3 to examine whether personalised nutrition advice was more effective than generalised dietary advice. Contrast 2 compared L1 with L2-L3 to test whether personalisation based on phenotypic or phenotypic plus genotypic information differed from that based on dietary assessment only. Contrast 3 compared L2 with L3 to test whether the addition of genotypic information promoted changes that differed from those using phenotypic and dietary information only.

Using the same approach described above, information from the dietary change questionnaire was used to examine whether perceived reduction in the amount of fat, sugar, salt, or a composite measure of these three, consumed over the 6-month intervention differed by intervention arm. Percentage concordance was estimated between the perceptions of reductions in intake of fat, sugar and salt (from the dietary change questionnaire) and measured decreases in intake of these nutrients from the FFQ (i.e. % change from total fat and sugars and grams/day of salt between baseline and month 6). For this purpose, the binary indicator for perception of dietary change for each nutrient (strongly agree/agree vs disagree/strongly disagree) was tabulated against a binary indictor for change in dietary intake for that nutrient (decrease in intake vs increase in intake).

## Results

A total of 1607 participants were randomised into the intervention and 1270 of these completed the intervention and were included in the present analysis (Additional file 4). A full description of the characteristics of individuals who dropped out is described elsewhere [44]. Briefly, attrition did not significantly differ between the control and personalised nutrition arms, yet females, younger adults and adults with obesity were more likely to drop out. Baseline characteristics of participants were comparable across control and intervention arms (Additional file 5). Overall, the mean age of participants was 40.9 (SD 13.0) years, 57% were female, 46% had overweight or obesity, 40% were in a professional or managerial occupation, 78% met physical activity recommendations and 12% were current smokers (Additional file 6). Participants with highest %E from discretionary foods and beverages (ADG and FSS) at baseline were on average younger, had overweight or obesity, had higher blood cholesterol concentrations and were under-reporters of energy intake (Additional file 6). Using the ADG classification, participants with highest %E from discretionary foods and beverages at baseline were on average male, in intermediate occupations, less physically active, more sedentary and using medication. There was no significant difference in smoking status by %E intake from discretionary foods.

**Fig. 1:**
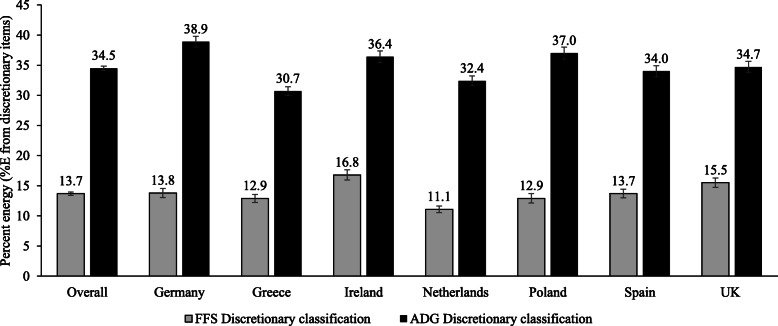
Intakes of discretionary foods and beverages (%E) at baseline for participants in seven European countries. Values represent means ± SE. FSS, Food Standards Scotland classification of discretionary foods and beverages. ADG, Australian Dietary Guidelines classification of discretionary foods and beverages

At baseline, 100 and 99.1% of participants consumed ADG and FSS discretionary items, respectively. The contribution of discretionary foods to intake of total energy and nutrients at baseline overall and by country is presented in Fig. 1. Overall, FSS discretionary foods contributed 13.7% of participants’ daily energy intake whereas when classified using the ADG, discretionary foods contributed 34.7% of daily energy intake. The positive correlation between both measures was strong (r = 0.67). When stratified by country, %E from FSS discretionary foods was highest in Ireland and lowest in the Netherlands (Fig. 1). In contrast, %E from ADG discretionary foods was highest in Germany and lowest in Greece.

### Three-month effects of personalised nutrition intervention

The effects of the intervention on intake of discretionary foods and beverages at month 3 are shown in Additional file 7. Using the FSS classification, there were no significant differences in %E from discretionary foods items between those randomised to personalised nutrition and to the control intervention. However, reductions in %E, % total fat, SFA and sugars from ADG discretionary foods and beverages were greater in participants randomised to receive personalised nutrition advice (combined L1–3) compared with the control. No significant differences between levels of personalised nutrition advice were identified.

The % contribution to intake of total sugars from sweets and snacks was lower in participants randomised to personalised nutrition compared with the control (Additional file 7) but there were no significant effects of personalised nutrition on the % contribution to intake of total fat, SFA and salt from sweets and snacks.

### Six-month effects of personalised nutrition intervention

The effects of the intervention on intake of discretionary foods and beverages at month 6 are shown in Table 1. Randomisation to personalised nutrition advice (combined L1–3) resulted in greater reductions in % sugars from FSS discretionary items compared with the control group but there were no significant differences between effects of levels of personalised nutrition advice (L1 to L3). Participants randomised to receive personalised nutrition advice (combined L1–3) reported larger reductions in %E, % total fat, SFA and sugars from ADG discretionary items compared with the control. When comparing personalised nutrition arms, reductions in %E, % total fat, SFA and salt were greater for those randomised to L3 compared with L2.

**Table 1:** Effect of personalised nutrition intervention on intake of discretionary foods and beverages at month 6

	Control Mean (L0)	Personalised nutrition Mean (L1, L2, L3)	Personalised nutrition			L0 vs (L1 + L2 + L3)	L1 vs (L2 + L3)	L2 vs L3
			L1	L2	L3			
n	312	958	312	325	321			
Discretionary intake, FSS								
Energy, % of total (kJ)	12.0 ± 0.41	11.1 ± 0.23	11.0 ± 0.41	11.6 ± 0.40	10.8 ± 0.41	0.06	0.68	0.14
Total fat, % of total (g/day)	13.5 ± 0.47	12.7 ± 0.27	12.5 ± 0.47	13.2 ± 0.46	12.2 ± 0.47	0.12	0.65	0.12
Saturated fat, % of total (g/day)	17.1 ± 0.58	16.0 ± 0.33	15.8 ± 0.58	16.7 ± 0.57	15.5 ± 0.57	0.10	0.69	0.16
Total sugars, % of total (g/day)	21.1 ± 0.65	19.0 ± 0.37	18.7 ± 0.65	19.6 ± 0.64	18.6 ± 0.64	0.005	0.58	0.31
Salt, % of total (g/day)	6.99 ± 0.32	6.53 ± 0.18	6.43 ± 0.32	6.87 ± 0.31	6.29 ± 0.31	0.21	0.69	0.19
Discretionary intake, ADG								
Energy, % of total (kJ)	32.7 ± 0.59	31.2 ± 0.59	31.0 ± 0.59	32.5 ± 0.58	30.1 ± 0.58	0.031	0.65	0.005
Total fat, % of total (g/day)	33.3 ± 0.65	31.5 ± 0.37	31.1 ± 0.65	32.7 ± 0.64	30.7 ± 0.64	0.021	0.43	0.028
Saturated fat, % of total (g/day)	37.8 ± 0.75	36.0 ± 0.43	35.5 ± 0.75	37.3 ± 0.74	35.0 ± 0.74	0.034	0.49	0.030
Total sugars, % of total (g/day)	34.7 ± 0.78	31.7 ± 0.44	31.4 ± 0.78	32.8 ± 0.76	30.9 ± 0.77	< 0.001	0.58	0.08
Salt, % of total (g/day)	36.6 ± 0.71	35.9 ± 0.41	35.8 ± 0.71	37.3 ± 0.69	34.6 ± 0.70	0.42	0.89	0.007
Contribution made by sweets and snacks, % of total intake (g/day)								
Total fat	16.3 ± 0.50	16.3 ± 0.28	16.2 ± 0.50	16.6 ± 0.49	16.1 ± 0.49	0.94	0.83	0.48
Saturated fat	18.5 ± 0.49	18.0 ± 0.33	17.8 ± 0.57	18.5 ± 0.56	17.8 ± 0.56	0.49	0.67	0.40
Total sugars	21.5 ± 0.63	19.1 ± 0.36	18.7 ± 0.63	19.3 ± 0.63	19.2 ± 0.63	< 0.001	0.51	0.91
Salt	7.45 ± 0.30	6.97 ± 0.17	6.99 ± 0.30	7.26 ± 0.30	6.65 ± 0.29	0.17	0.93	0.14

Values represent adjusted means ± SE; contrast analyses were used to test for significant differences between groups; ancova were adjusted for baseline intake (for month 6 analyses), age, sex and country. FSS, Food Standards Scotland classification of discretionary foods and beverages. ADG, Australian Dietary Guidelines classification of discretionary foods and beverages. L0, Level 0 - Control, generalised advice; L1, Level 1 – personalised advice based on diet alone; L2, Level 2 – personalised advice based on diet and phenotype; L3, Level 3 – personalised advice based on diet, phenotype and genotype

At month 6, the % contribution to intake of total sugars from sweets and snacks was lower in participants randomised to personalised nutrition compared with the control (Table 1) but there were no significant differences between levels of personalised nutrition advice. Personalised nutrition did not affect the contribution (%) made by sweets and snacks to intake of total fat, SFA and salt.

### Perceptions of change in intakes of fat, sugar and salt

A total of 62, 54 and 59% of participants agreed that they had reduced their intake of total fat, sugars and salt, respectively. More participants randomised to receive personalised nutrition advice indicated they had reduced their intake of total fat, sugars and salt compared with the control (Additional file 8). Concordance between perceptions reported in the dietary change questionnaire and estimates of dietary intake from the FFQ for reduced intake of total fat, sugars and salt was 56.8, 44.6 and 71.0%, respectively.

## Discussion

This study examined changes in intake of discretionary foods and beverages following a personalised nutrition intervention among European adults participating in the Food4Me Study. We estimated intakes of energy and nutrients using two different discretionary food classification systems – the more inclusive ADG classification that included all foods high in fat, added sugars and salt and the more restrictive FSS classification [3, 4]. Our main findings were that personalised nutrition advice reduced the contribution of discretionary foods and beverages to intake of energy, total fat, SFA and total sugars compared with generalised dietary advice, although this was most evident when discretionary foods were defined using the more inclusive ADG classification. Our results illustrate the importance of developing a consensus in how to classify discretionary foods and beverages. The Food4Me personalised nutrition intervention was designed to improve overall diet but was not designed specifically to reduce intakes of discretionary foods and beverages. Thus, these findings highlight opportunities for future personalised nutrition interventions to target these food items to achieve even greater dietary changes with potentially greater health advantages.

The present analysis provides insights into the consumers of discretionary food items across Europe. Confirming previous research, we observed that discretionary food intake at baseline differed by participant characteristics. European studies have shown that intake of foods high in saturated fat, added sugar and salt and sugar-containing beverages are greater in younger adults who, in general, have lower quality diets than older adults [45]. Our findings of higher blood cholesterol concentration and greater adiposity among participants with greater intake of discretionary foods and beverages are consistent with previous research, where greater intake of discretionary foods has been linked to poorer cardiometabolic health [46] and higher BMI [47].

Our comparison of two discretionary classifications has important implications for the estimation of percentage energy from these items. Comparing intake from these two classifications, the difference in %E was more than two-fold higher using the ADG classification. Consistent with our results, application of the FSS discretionary classification to Scottish national nutrition data indicated that 19% of energy came from discretionary items [4], while data from the 2011–2012 Australian Health Survey indicated that the %E from discretionary items in adults was 33% [48]. National survey data from Mexico estimates that discretionary items contribute 26% of energy intake in adults [10], although the classification of discretionary items used did not include processed meats. Given the role of food processing in increasing the content of fat, sugar and salt in foods globally [49, 50], there is clearly overlap between research on discretionary foods and that on (ultra) processed foods. With high added sugars, fats and salt foods being key components targeted for reduction in dietary guidelines globally, differences in classification of discretionary foods may have a significant impact on the interpretation, and implementation, of public health policies.

An important difference between the two discretionary classification used relates to whether foods consumed as part of a meal should be included in discretionary items. In the FSS, foods high in saturated fat, added sugars and salt, such as processed meats, that were consumed typically as part of a meal, were considered non-discretionary because they contributed to the intake of nutrients, such as protein and iron [4]. Given that many high fat, salt and added-sugars foods and beverages that are consumed as part of a meal can be substituted for healthier alternatives, it would be prudent for these items to be considered in the classification of discretionary foods. Including these foods high in fat, salt and added-sugars and sugar-rich beverages may aid the design of more effective dietary interventions.

The effectiveness of the present personalised nutrition intervention on reducing discretionary food intake may be due partly to the design of messaging used within the Food4Me study. Advice was based on behaviour change taxonomies [30] and included strategies such as goal setting, swapping strategies when shopping and cooking tips. There was some evidence that personalised nutrition advice that included genetic information was more effective than personalised nutrition advice based on diet and phenotype information only, possibly because two of the genotypes used in the intervention are influenced by fat intake (apolipoprotein E (*APOE*) and transcription factor 7-like 2 (*TCF7L2*)) [23, 51]. However, the magnitude of reductions in %E from discretionary foods were comparable between personalised nutrition advice based on diet alone. This suggests that the added participant burden and expense of higher-level personalisation may not add clinically significant benefit and warrants further research to determine the most effective basis for personalisation. Individual participants were given advice based on their priority nutrients and food groups. Since total fat, SFA and salt were key target nutrients, messaging recommended limiting foods and beverages high in fat and salt when intake of these nutrients were above recommendations. However, the Food4Me Study did not focus on discretionary foods and beverages per se and did not include added sugars as a target nutrient.

Recent evidence from the 2011–2012 Australian Health Survey found a significantly higher degree of under-reporting of discretionary foods compared with other food groups, leading the authors to conclude that low energy-reporting is likely to over-estimate diet quality [52]. Similarly, in the Food4Me Study, although estimates of misreporting (over- and under-reporting) of dietary energy intake were similar across all quartiles of discretionary food intake, estimates of under-reporting of dietary energy intake and the proportion of overweight /obese participants were higher among participants reporting greater intake of discretionary foods. Further personalised nutrition research is thus needed to examine whether a greater focus on discretionary items would result in greater improvements in the nutritional quality of dietary intake independent of differential energy under-reporting. Moreover, participants in the Food4Me study reduced their intake of total energy intake as a result of the personalised nutrition intervention [24] and future research should consider which food swapping strategies are adopted by participants, and the extent to which participants chose to reduce their portion sizes.

Our findings for change in %E from discretionary items estimated from the FFQ were supported by data from the perceptions of dietary change questionnaire. There were positive associations between reported change in intake of discretionary foods and in perceptions of change in intake of total fat, sugar and salt. Although both questionnaires are self-reported and subject to social desirability biases [53], similarity in findings between measures suggests that perceived dietary behaviour change mirrored change in reported intake from an FFQ. Studies have shown concordance between dietary intake and other dietary indictors, such as food liking [54], however few have examined comparability with dietary change questionnaires. These findings warrant further investigation to determine the concordance between measures of discretionary intake and future research should combine the use of these subjective measures with objectively measured indicators of health.

A limitation of our study is that data were self-measured and self-reported via the internet. Although the FFQ has been validated against a 4-day weighed food record, all dietary intake assessments are subject to misreporting biases [34]. The level of detail in the 157 FFQ items was insufficient to accurately classify all foods and beverages according to the ADG discretionary measure, which were based on a 24-h recall. Nonetheless, the FSS classification framework for discretionary foods and beverages was more generic and so less likely to be impacted by misclassification of food and beverage items. Information on intake of free sugars was not available, and so the percentage energy from total sugars includes sugars from non-discretionary sources, such as whole fruit, and should be interpreted with this in mind. Moreover, added sugars was not a target nutrient for personalised advice. The accuracy of internet-based, self-reported anthropometric data from the Food4Me Study have been reported previously [42]. Since 97% of Food4Me participants were Caucasian, research in wider ethnicity groups is required to generalise our findings to other populations. Moreover, our sample is a self-selected group of individuals who may be more health-conscious than the general population and who may be more motivated to improve their health behaviours. Nonetheless, the characteristics of participants who registered interest in joining the study were similar to the wider population of European adults, who would benefit from improved diet and more physical activity [55]. Future research is needed in harder to reach population groups, such as young males and those experience socio-economic disadvantage, to understand whether personalised behaviour change interventions can achieve change in such population groups. While analyses were adjusted for appropriate confounders, we cannot discount the possibility of residual confounding. As we did not account for multiple testing, the risk of type 1 error is higher and so results should be interpreted with this in mind. Lastly, the Food4Me intervention study was not double-blind and thus may be subject to reactivity bias. However, participants in all treatment groups completed the same measures (including FFQ, buccal swabs and blood spot cards) so that it is unlikely that differences between treatment groups, as reported here, can be attributed to reactivity bias.

This study had a number of strengths. The Food4Me study is the largest RCT on the effectiveness of personalised nutrition advice in European adults to date. In the absence of a European classification of “discretionary food and beverages”, we implemented two contrasting measures (the ADG that was more inclusive and the FSS that was more restrictive) for estimating the %E from discretionary foods from a validated FFQ. These two measures allowed for a comparison between country-specific guidelines for the classification of discretionary foods, providing insights into the implications of a more limited and more extensive classification of these foods. Inclusion of dietary intake derived from an FFQ as well as a dietary change questionnaire provided behavioural insights into concordance of perceived and measured dietary changes.

## Conclusions

The present study shows that, following a 6-month intervention, personalised nutrition advice reduced the contribution of discretionary foods and beverages to intake of total energy and of fat, sugar and salt. Importantly, apart from %E from sugars, there was only evidence of an intervention effect when the discretionary classification included all high fat, added sugars and salt foods. Participants randomised to personalised nutrition advice also perceived that they had reduced the amounts of fat, sugar and salt in their diet, which may be attributable to higher perceived self-efficacy for healthy eating and may result in greater potential for sustained dietary improvement [56]. These results have implications for the design of future personalised nutrition interventions with greater focus on intake of discretionary foods and beverages. Considering the global political focus on discretionary foods and beverages as targets for fat, sugar and salt taxes [57], this study provides policy-relevant insights into the pan-European contribution of these foods to energy intake. Further policy implications of this study include highlighting the importance of gaining a consensus on the classification of discretionary foods and beverages and the use of food-based messaging for consumers that consider the eating context of the foods consumed.

## Supplementary Information

**Additional file 1.** CONSORT checklist.

**Additional file 2.** TIDieR checklist.

**Additional file 3.** Classification of discretionary foods and beverages according to Food Standards Scotland (FSS) and the Australian Dietary Guidelines (ADG) used in the present analysis.

**Additional file 4.** Consort diagram. Consort diagram of participants included in the Food4Me study.

**Additional file 5.** Baseline characteristics of participants according to control and intervention arms.

**Additional file 6.** Characteristics of participants according to baseline intake of discretionary foods and beverages according to the Food Standards Scotland (FSS) classification.

**Additional file 7.** Effect of personalised nutrition intervention on intake of discretionary foods and beverages at month 3.

**Additional file 8.** Effect of personalised nutrition intervention on proportion of participants who perceived that they reduced the amount of total fat, sugars and salt consumed over the 6 months intervention.

## Data Availability

The datasets used and/or analysed during the current study are available from the corresponding author on reasonable request.

## Abbreviations

ADG: Australian Dietary Guidelines
BMI: Body mass index
FFQ: Food frequency questionnaire
FSS: Food Standards Scotland
RCT: Randomised controlled trial
WC: Waist circumference
