# Supplementary material for: Personalised nutrition advice reduces intake of discretionary foods and beverages: findings from the Food4Me randomised controlled trial
Source: Int J Behav Nutr Phys Act. 2021 Jun 7;18:70. doi: 10.1186/s12966-021-01136-5 (PMC8183081; doi:10.1186/s12966-021-01136-5)
Supplement: Supplementary file 3 — Additional file 3. Classification of discretionary foods and beverages according to Food Standards Scotland (FSS) and the Australian Dietary Guidelines (ADG) used in the present analysis. [file 12966_2021_1136_MOESM3_ESM.docx]

**Additional file 3.** Classification of discretionary foods and beverages according to Food Standards Scotland (FSS) and the Australian Dietary Guidelines (ADG) used in the present analysis

| **Discretionary classification^1^** | | **Food4me FFQ item** | **Included in analysis** | |
| --- | --- | --- | --- | --- |
| **Food group** | **Food items included** |  | **FSS** | **ADG** |
| Savoury biscuits | Savoury biscuits | Cream crackers, cheese biscuits, rusks | ✕ | ✓ |
| Potato products | Chips | Chips | ✕ | ✓ |
| Pizza or snack product | Pizza, spring rolls and dumplings | Pizza, calzone | ✕ | ✓ |
|  |  | Spring rolls | ✕ | ✓ |
|  |  | Potato or plain dumplings | ✕ | ✓ |
| Processed meat, battered fish, sausages, burgers | Processed meat, sausages and burgers | Burgers e.g. Beef, meatballs | ✕ | ✓ |
|  |  | Processed chicken or poultry e.g. nuggets, goujons, fried | ✕ | ✓ |
|  |  | Bacon | ✕ | ✓ |
|  |  | Sliced cold meats e.g. ham, turkey | ✕ | ✓ |
|  |  | Cured meats e.g. corned beef, salami, chorizo | ✕ | ✓ |
|  |  | Sausages e.g. pork, jadwurst | ✕ | ✓ |
|  |  | Savory pies, meat pies, pasties, sausage rolls | ✕ | ✓ |
|  |  | Pate e.g. meat or liver | ✕ | ✓ |
|  |  | Fried fish in batter | ✕ | ✓ |
|  |  | Fish fingers, fish cakes | ✕ | ✓ |
| Cream | Cream | Double/clotted cream | ✕ | ✓ |
| Pastry | Quiche | Quiche, savoury pancakes | ✕ | ✓ |
| Salad dressing | Salad dressings (including french-style and excluding vinegar) | Salad cream, mayonnaise | ✕ | ✓ |
|  |  | French dressing, vinaigrette | ✕ | ✓ |
| Butter and cooking margarine | Butter and cooking margarine | Butter | ✕ | ✓ |
|  |  | Block/hard margarine e.g. stork/krona | ✕ | ✓ |
| Sweet biscuits | Sweet biscuits (not chocolate) and cereal bars, chocolate biscuits, fully-coated chocolate biscuits or wafers, sweet biscuits including half-coated chocolate biscuits, cereal bars and cereal based cakes. | Sweet biscuits, chocolate e.g. digestive, cookies | ✓ | ✓ |
|  |  | Sweet biscuits, plain e.g. nice, ginger | ✓ | ✓ |
| Cakes, pastries and puddings | Danish pastries, pecan danish, fruit pies, fruit tarts, jam tarts, custard tart, treacle tart, flans, bakewells, chorley cakes, tortes, egg custards, raisin & currant puffs, fruit pastries, sponge cakes, gateau, stollen, parkin, swiss rolls, chocolate cakes, cream cakes, éclairs, chocolate croissant, pain au chocolat, chocolate brioche, meringues, pavlova, pop tarts, doughnuts, american muffin ‘cakes’, blueberry muffins, chocolate muffins, frozen cheesecakes, frozen chocolate filled pancakes, frozen eclairs, frozen sponges and gateaux, (including those with ice-cream), frozen danish, frozen custard slice, frozen apple pie, frozen fruit pies, frozen pavlova, frozen profiteroles. Instant/dessert whips, trifle mixes, cheesecake mixes, crumble mix, fruit puddings, summer fruit pudding, sponge puddings, chocolate sponge pudding, treacle sponge pudding, syrup puddings, fruit fritters, christmas pudding, bread pudding, sticky toffee pudding | Plain cakes e.g. fruit, sponge, scones, gingerbread | ✓ | ✓ |
|  |  | Rich cakes e.g. chocolate, cheesecake | ✓ | ✓ |
|  |  | Flapjacks, muesli bars, oatmeal cookies | ✓ | ✓ |
|  |  | Buns, muffins, pastries e.g. croissants, doughnuts | ✓ | ✓ |
|  |  | Waffles, pancakes, crepes | ✓ | ✓ |
|  |  | Fruit pies, tarts, crumbles | ✓ | ✓ |
|  |  | Sponge puddings | ✓ | ✓ |
|  |  | Milk puddings, e.g. rice, custard, trifle | ✓ | ✓ |
| Ice cream and dairy desserts | Ice cream tub or block, ice cream cornets, choc-ices, lollies with ice cream, ice lollies, sorbets, chilled dairy desserts including mousse, pannacotta, cheesecake, trifle, syllabub, fruit fool, tiramisu, twin pots, frozen yoghurt, takeaway milkshakes | Ice-cream, choc ices | ✓ | ✓ |
|  |  | Sorbets and jellies | ✓ | ✓ |
| Confectionery | Solid chocolate bars, filled chocolate-coated bars, sweets, mints, boiled sweets, fudges, toffees, caramels, jellies and unspecified 'sweets,' uncoated toffee or fudge, chocolate éclairs, caramels, pick 'n' mix, nougat, liquorice and other sweets | Chocolates, single or squares | ✓ | ✓ |
|  |  | Chocolate snack bars e.g. mars/crunchie | ✓ | ✓ |
|  |  | Sweets, toffees, mints, liquorice | ✓ | ✓ |
| Crisps and savoury snacks | Crisps and potato snacks, cereal snacks, popcorn, poppadums, prawn crackers, corn snacks (based on maize) and wheat based savoury snacks | Crisps or other packet snacks eg wotsits | ✓ | ✓ |
| Pastry or snack product | Pastry and snack products | Baklava, kantaifi | ✓ | ✓ |
|  |  | Snackbarproducts eg frikandel, kroket, bamibal, kaassouflé | ✓ | ✓ |
|  |  | Stroopwaffel | ✓ | ✓ |
| Gravy and savoury sauces | Sauces (excluding homemade tomato sauce and non-creamy sauces) | Creamy soups e.g. chowder, cream of mushroom | ✕ | ✓ |
|  |  | Creamy sauces e.g. carbonara/cheese | ✕ | ✓ |
|  |  | Dark sauces e.g. gravy, stir-fry sauces | ✕ | ✓ |
|  |  | Tomato sauces e.g. in bolognese, on meatballs or pasta | ✕ | ✓ |
|  |  | Tomato ketchup | ✕ | ✓ |
| Pickles | Pickles, chutney and sate | Pickles, chutney, satésaus (saté) | ✕ | ✓ |
| Jams and spreads | Jam and chocolate or yeast spreads | Marmite/bovril | ✕ | ✓ |
|  |  | Jam/marmalade/honey | ✕ | ✓ |
|  |  | Nut or chocolate spreads e.g. peanut butter, nutella | ✕ | ✓ |
| Sugar | Added sugar | Sugar, added to tea, coffee, cereal | ✕ | ✓ |
| Beverage | Hot chocolate base made up with water | Hot chocolate, ovaltine, horlicks, made with water | ✕ | ✓ |
| Low calorie soft drinks | Low calorie soft drinks, including intense sweetened drinks | Low calorie/ diet fizzy soft drinks | ✕ | ✓ |
| Sugar containing soft drinks | Soft drinks, concentrated, not low calorie, soft drinks, not concentrated, not low calorie, soft drink (incl carbonates & still) - not low calorie (including drinks where calorie content unspecified), soft drink where pure juice or juice drink not specified, mixer recorded with spirits and alcopops. | Fizzy soft drinks e.g. coca cola / lemonade | ✓ | ✓ |
|  |  | Fruit squash/ cordial / nectar | ✓ | ✓ |
|  |  | Sweet alcoholic drinks, e.g. alcopops, cocktails | ✓ | ✓ |
| Alcoholic beverages | Wine, beer, port and spirits | Wine | ✕ | ✓ |
|  |  | Beer, larger, cider | ✕ | ✓ |
|  |  | Port, sherry, vermouth, liqueurs | ✕ | ✓ |
|  |  | Spirits, e.g. gin, brandy, whiskey, vodka | ✕ | ✓ |

1. The discretionary classification columns use terminology from both the FSS and ADG classifications for discretionary foods to provide a summary of the food groups and descriptions. For FSS discretionary items, the food groups and descriptions are worded exactly as included in Appendix 1 of the FSS discretionary classification. For ADG discretionary items, the food groups and descriptions are paraphrased.

FSS, Food Standards Scotland classification of discretionary items; ADG, Australian Dietary Guidelines classification of discretionary item.

✓ indicates that the Food4Me food item was included in the discretionary measure used in the present study
