# Supplementary material for: Personalised nutrition advice reduces intake of discretionary foods and beverages: findings from the Food4Me randomised controlled trial
Source: Int J Behav Nutr Phys Act. 2021 Jun 7;18:70. doi: 10.1186/s12966-021-01136-5 (PMC8183081; doi:10.1186/s12966-021-01136-5)
Supplement: Supplementary file 4 — Additional file 4. Consort diagram. Consort diagram of participants included in the Food4Me study. [file 12966_2021_1136_MOESM4_ESM.docx]

Level 0 “Control”

n=387

Level 1

n=414

Level 2

n=404

Level 3

n=402

Completed the study

n=312

Completed the study

n=325

Completed the study

n=321

Dropouts immediately after randomization

n=27

Dropouts immediately after randomization

n=41

Dropouts immediately after randomization

n=28

Dropouts immediately after randomization

n=31

Lost to follow up

n=48

Lost to follow up

n=61

Lost to follow up

n=53

Lost to follow up

n=50

Completed the study

n=312

Participants who registered online for the Food4Me Study

n=5562

Participants randomized into one of the 4 arms on the intervention n=1607

**Excluded, n=1631***

- Not willing to share information, n=35

- Incomplete 2^nd^ screening questionnaire, n =562

- Pregnant, n=181

- Therapeutic diet, n=350

- Food allergy/intolerance, n=658

- No Internet, n=28

2^nd^ Screening questionnaire

n=3811

1^st^ Screening questionnaire

n=5442

**Excluded, n=120**

- Incomplete 1^st^ screening questionnaire

**Excluded, n= 1029***

- Second consent not given, n=238

- Incomplete/under-reported food frequency questionnaire, n=535

- Food allergy/intolerance, n=93

- Therapeutic diet, n=199

- Limited physical activity n=252

**Excluded, n=1175**

- Study design and sample size estimation required n=1607 only

**Analysis**

**Follow-up**

**Allocation**

**Enrolment**

**Additional file 4.** Consort diagram of participants included in the Food4Me study
