# Supplementary material for: Personalised nutrition advice reduces intake of discretionary foods and beverages: findings from the Food4Me randomised controlled trial
Source: Int J Behav Nutr Phys Act. 2021 Jun 7;18:70. doi: 10.1186/s12966-021-01136-5 (PMC8183081; doi:10.1186/s12966-021-01136-5)
Supplement: Supplementary file 5 — Additional file 5. Baseline characteristics of participants according to control and intervention arms. [file 12966_2021_1136_MOESM5_ESM.docx]

**Additional file 5.** Baseline characteristics of participants across control and intervention arms

|  | **Control (n=312)** | **Personalised nutrition intervention arms** | | |
| --- | --- | --- | --- | --- |
|  |  | **L1 (n=312)** | **L2 (n=325)** | **L3 (n=321)** |
| **Demographics** | | | |  |
| Age, years | 40.5 (13.0) | 40.7 (12.9) | 40.9 (12.7) | 41.3 (13.3) |
| Female, % | 57.4 | 55.7 | 58.2 | 56.7 |
| Occupation, % |  |  |  |  |
| Professional and managerial | 39.1 | 38.5 | 41.5 | 40.8 |
| Intermediate occupations | 24.7 | 27.6 | 26.5 | 25.9 |
| Routine and manual | 10.3 | 8.65 | 9.23 | 9.97 |
| **Anthropometrics** |  |  |  |  |
| Body weight, kg | 74.1 (15.3) | 73.8 (16.2) | 75.1 (15.7) | 75.6 (15.7) |
| BMI, kg/m^2^ | 25.1 (4.45) | 25.0 (4.70) | 25.7 (4.96) | 25.7 (4.89) |
| Overweight/obese, % | 46.8 | 40.7 | 45.9 | 50.2 |
| Waist circumference, cm | 85.6 (13.5) | 84.6 (13.7) | 86.3 (14.1) | 87.0 (13.4) |
| **Physical activity** | | | |  |
| Physical activity level | 1.71 (0.18) | 1.76 (0.21) | 1.73 (0.16) | 1.73 (0.17) |
| MVPA, min/d | 42.2 (29.8) | 50.0 (32.2) | 44.1 (28.3) | 43.8 (30.5) |
| Meet recommendations, % | 73.0 | 81.9 | 77.7 | 77.4 |
| Sedentary behaviour, min/d | 749 (75.1) | 738 (72.9) | 748 (75.0) | 753 (77.5) |
| **Health and disease history** |  |  |  |  |
| Total blood cholesterol, mmol/L | 4.62 (0.91) | 4.60 (0.92) | 4.60 (0.96) | 4.61 (0.95) |
| Medication use, % | 31.7 | 26.6 | 32.0 | 31.5 |
| Current smoker, % | 14.1 | 10.9 | 9.23 | 12.8 |

Values represent means (SD) or percentages. L=Level; L1, Participants received personalised nutrition advice based on their current diet; L2, Participants received personalised nutrition advice based on their current diet and phenotype; L3, Participants received personalised nutrition advice based on their current diet, phenotype and genotype; MVPA, Moderate to vigorous physical activity
