# Supplementary material for: Personalised nutrition advice reduces intake of discretionary foods and beverages: findings from the Food4Me randomised controlled trial
Source: Int J Behav Nutr Phys Act. 2021 Jun 7;18:70. doi: 10.1186/s12966-021-01136-5 (PMC8183081; doi:10.1186/s12966-021-01136-5)
Supplement: Supplementary file 6 — Additional file 6. Characteristics of participants according to baseline intake of discretionary foods and beverages according to the Food Standards Scotland (FSS) classification. [file 12966_2021_1136_MOESM6_ESM.docx]

**Additional file 6.** Characteristics of participants according to baseline intake of discretionary foods and beverages according to the Food Standards Scotland (FSS) classification^1^

|  | **All (n=1270)** | **Percentage total energy intake from FSS discretionary foods** | | **P trend** |
| --- | --- | --- | --- | --- |
|  |  | **Q1 (n=318)** | **Q4 (n=317)** |  |
| Discretionary food, %E | 13.7 (9.93) | 4.02 (1.99) | 27.4 (9.16) | **<0.001** |
| Energy intake misreporters, % | 21.0 | 24.5 | 23.0 | 0.28 |
| **Demographics** | | | |  |
| Age, years | 40.9 (13.0) | 43.2 (13.7) | 40.0 (12.0) | **0.004** |
| Female, % | 57.4 | 55.7 | 60.6 | 0.22 |
| Occupation, % |  |  |  |  |
| Professional and managerial | 40.0 | 39.9 | 41.3 | 0.72 |
| Intermediate occupations | 26.1 | 24.5 | 27.4 | 0.44 |
| Routine and manual | 9.53 | 10.1 | 9.15 | 0.67 |
| **Anthropometrics** |  |  |  |  |
| Body weight, kg | 74.6 (15.7) | 74.3 (15.8) | 76.6 (16.8) | **0.004** |
| BMI, kg/m^2^ | 25.4 (4.76) | 25.2 (5.02) | 26.2 (4.90) | **<0.001** |
| Overweight/obese, % | 45.9 | 41.5 | 53.9 | **<0.001** |
| Waist circumference, cm | 85.9 (13.7) | 85.2 (13.7) | 87.5 (14.4) | **<0.001** |
| **Physical activity** | | | |  |
| Physical activity level | 1.73 (0.18) | 1.74 (0.18) | 1.73 (0.19) | 0.53 |
| MVPA, min/d | 45.0 (30.3) | 44.4 (30.6) | 43.4 (29.1) | 0.90 |
| Meet recommendations, % | 77.5 | 76.0 | 74.2 | 0.62 |
| Sedentary behaviour, min/d | 747 (75.2) | 750 (75.4) | 744 (75.7) | 0.25 |
| **Health and disease history** |  |  |  |  |
| Total blood cholesterol, mmol/L | 4.65 (0.94) | 4.65 (0.93) | 4.67 (1.02) | **0.046** |
| Medication use, % | 30.5 | 30.2 | 33.4 | 0.35 |
| Current smoker, % | 11.7 | 12.0 | 12.9 | 0.92 |

Values represent means (SD) or percentages. L=Level; L1, Participants received personalised nutrition advice based on their current diet; L2, Participants received personalised nutrition advice based on their current diet and phenotype; L3, Participants received personalised nutrition advice based on their current diet, phenotype and genotype; MVPA, Moderate to vigorous physical activity

1, Multiple linear regression was used to test for differences in characteristics (dependant variable) by quartiles of discretionary food and beverage intake (independent variable). Analyses were adjusted for age, sex, country and intervention arm. Physical activity level, MVPA and sedentary behaviour were additionally adjusted for time wearing the accelerometer and season. Energy misreporting was estimated as a binary variable (yes, no): under-reporting was operationalized as energy intake less than basal metabolic rate*1.1 (25), where basal metabolic rate was calculated according to the Oxford equation (26) and over-reporting as more than 4500 kcal/day (27). Data on Physical activity level, MVPA and sedentary time were available in n=1147 participants
