# Supplementary material for: Personalised nutrition advice reduces intake of discretionary foods and beverages: findings from the Food4Me randomised controlled trial
Source: Int J Behav Nutr Phys Act. 2021 Jun 7;18:70. doi: 10.1186/s12966-021-01136-5 (PMC8183081; doi:10.1186/s12966-021-01136-5)
Supplement: Supplementary file 7 — Additional file 7. Effect of personalised nutrition intervention on intake of discretionary foods and beverages at month 3. [file 12966_2021_1136_MOESM7_ESM.docx]

**Additional file 7.** Effect of personalised nutrition intervention on intakes of discretionary foods and beverages at month 3

|  | **Control**  **Mean**  **(L0)** | **Personalised nutrition**  **Mean**  **(L1, L2, L3)** | **Personalised nutrition** | | | **L0**  **vs (L1+L2+L3)** | **L1**  **vs**  **(L2+L3)** | **L2**  **vs**  **L3** |
| --- | --- | --- | --- | --- | --- | --- | --- | --- |
|  |  |  | **L1** | **L2** | **L3** |  |  |  |
| n | 312 | 958 | 312 | 325 | 321 |  |  |  |
| Discretionary intake, FSS |  |  |  |  |  |  |  |  |
| Energy, % of total (kJ) | 12.3 ± 0.42 | 11.5 ± 0.24 | 11.3 ± 0.41 | 12.1 ± 0.41 | 11.2 ± 0.41 | 0.10 | 0.49 | 0.15 |
| Total fat, % of total (g/day) | 13.8 ± 0.46 | 12.9 ± 0.26 | 12.4 ± 0.46 | 13.4 ± 0.45 | 12.7 ± 0.46 | 0.08 | 0.28 | 0.33 |
| Saturated fat, % of total (g/day) | 17.4 ± 0.58 | 16.5 ± 0.33 | 16.1 ± 0.57 | 17.0 ± 0.56 | 16.5 ± 0.57 | 0.21 | 0.38 | 0.49 |
| Total sugars, % of total (g/day) | 21.3 ± 0.65 | 19.9 ± 0.37 | 19.5 ± 0.65 | 21.0 ± 0.64 | 19.2 ± 0.64 | 0.06 | 0.45 | 0.05 |
| Salt, % of total (g/day) | 7.21 ± 0.32 | 6.66 ± 0.18 | 6.98 ± 0.31 | 6.98 ± 0.31 | 6.49 ± 0.31 | 0.14 | 0.56 | 0.27 |
| Discretionary intake, ADG |  |  |  |  |  |  |  |  |
| Energy, % of total (kJ) | 33.6 ± 0.57 | 31.3 ± 0.32 | 31.1 ± 0.57 | 32.0 ± 0.56 | 30.8 ± 0.56 | **0.004** | 0.62 | 0.12 |
| Total fat, % of total (g/day) | 34.0 ± 0.63 | 31.2 ± 0.36 | 30.6 ± 0.62 | 32.1 ± 0.61 | 31.0 ± 0.61 | **<0.001** | 0.20 | 0.18 |
| Saturated fat, % of total (g/day) | 38.5 ± 0.71 | 35.4 ± 0.40 | 34.6 ± 0.71 | 36.6 ± 0.70 | 35.1 ± 0.70 | **<0.001** | 0.14 | 0.15 |
| Total sugars, % of total (g/day) | 34.3 ± 0.74 | 32.1 ± 0.42 | 31.8 ± 0.74 | 33.2 ± 0.72 | 31.4 ± 0.73 | **0.011** | 0.59 | 0.07 |
| Salt, % of total (g/day) | 36.6 ± 0.68 | 35.3 ± 0.38 | 35.2 ± 0.67 | 35.9 ± 0.66 | 34.9 ± 0.66 | 0.08 | 0.77 | 0.28 |
| Contribution made by sweets and snacks, % of total intake (g/day) |  |  |  |  |  |  |  |  |
| Total fat | 16.7 ± 0.50 | 16.4 ± 0.29 | 16.3 ± 0.50 | 16.7 ± 0.49 | 16.1 ± 0.50 | 0.55 | 0.86 | 0.42 |
| Saturated fat | 18.9 ± 0.58 | 18.5 ± 0.33 | 18.3 ± 0.58 | 18.9 ± 0.57 | 18.2 ± 0.57 | 0.55 | 0.65 | 0.42 |
| Total sugars | 21.8 ± 0.64 | 19.1 ± 0.36 | 19.1 ± 0.63 | 20.6 ± 0.62 | 19.3 ± 0.62 | **0.003** | 0.30 | 0.13 |
| Salt | 7.53 ± 0.30 | 7.14 ± 0.17 | 6.98 ± 0.30 | 7.31 ± 0.29 | 7.12 ± 0.29 | 0.26 | 0.50 | 0.14 |

Values represent adjusted means ± SE; contrast analyses were used to test for significant differences between groups; ancova were adjusted for baseline intake (for month 3 analyses), age, sex and country. FSS, Food Standards Scotland classification of discretionary foods and beverages. ADG, Australian Dietary Guidelines classification of discretionary foods and beverages. L0, Level 0 - Control, generalized advice; L1, Level 1 – personalised advice based on diet alone; L2, Level 2 – personalised advice based on diet and phenotype; L3, Level 3 – personalised advice based on diet, phenotype and genotype.
