# Supplementary material for: Personalised nutrition advice reduces intake of discretionary foods and beverages: findings from the Food4Me randomised controlled trial
Source: Int J Behav Nutr Phys Act. 2021 Jun 7;18:70. doi: 10.1186/s12966-021-01136-5 (PMC8183081; doi:10.1186/s12966-021-01136-5)
Supplement: Supplementary file 8 — Additional file 8. Effect of personalised nutrition intervention on proportion of participants who perceived that they reduced the amount of total fat, sugars and salt consumed over the 6 months intervention. [file 12966_2021_1136_MOESM8_ESM.docx]

**Additional file 8.** Effect of personalised nutrition intervention on proportion of participants who perceived that they reduced the amount of total fat, sugars and salt consumed over the 6 months intervention

|  | **Control**  **Mean (L0)** | **Personalised nutrition**  **Mean**  **(L1, L2, L3)** | **Personalised nutrition** | | | **L0**  **vs (L1+L2+L3)** | **L1**  **vs**  **(L2+L3)** | **L2**  **vs**  **L3** |
| --- | --- | --- | --- | --- | --- | --- | --- | --- |
|  |  |  | **L1** | **L2** | **L3** |  |  |  |
| n at baseline | 304 | 934 | 302 | 318 | 314 |  |  |  |
| Total fat (%) | 45.4 ± 26.8 | 67.4 ± 1.53 | 64.3 ± 2.68 | 64.5 ± 2.61 | 73.2 ± 2.63 | **<0.001** | 0.17 | **0.020** |
| Total sugars (%) | 48.0 ± 2.83 | 55.6 ± 1.61 | 48.1 ± 2.83 | 57.4 ± 2.75 | 60.9 ± 2.77 | **0.020** | **0.001** | 0.37 |
| Salt (%) | 39.7 ± 2.73 | 65.1 ± 1.56 | 66.5 ± 2.72 | 61.6 ± 2.66 | 67.1 ± 2.67 | **<0.001** | 0.51 | 0.15 |

Values represent adjusted means ± SE; contrast analyses were used to test for significant differences between groups; ancova were adjusted for baseline age, sex and country; the sample available for this analysis was n=1238 due to missing data in the dietary change questionnaire. L0, Level 0 - Control, generalised advice; L1, Level 1 – personalised advice based on diet alone; L2, Level 2 – personalised advice based on diet and phenotype; L3, Level 3 – personalised advice based on diet, phenotype and genotype.
